# Endocrinology application of molecular imaging: current role of PET/CT

**DOI:** 10.1007/s40618-024-02400-8

**Published:** 2024-06-05

**Authors:** L. Calderoni, L. Giovanella, S. Fanti

**Affiliations:** 1 Nuclear Medicine Division, IRCCS Azienda Ospedaliero-Universitaria di Bologna, Policlinico di S. Orsola, Via Albertoni 15, Bologna, Italy; 2 Department of Medical and Surgical Sciences (DIMEC), Alma Mater Studiorum University of Bologna, 40126 Bologna, Italy; 3 Clinic for Nuclear Medicine and Molecular Imaging, Imaging Institute of Southern Switzerland, Ente Ospedaliero Cantonale, Bellinzona, Switzerland; 4 Clinic for Nuclear Medicine, University Hospital and University of Zurich, Zurich, Switzerland

**Keywords:** PET/CT, [^11^C]Choline, [^18^F]F-choline, [C_1_-^11^C]Methionine, 2-[^18^F]FDG, 6-[^18^F]FDOPA, Endocrine tumors

## Abstract

**Background:**

In recent years, nuclear medicine imaging methods have proven to be of paramount importance in a wide variety of diseases, particularly in oncology, where they are crucial for assessing the extent of disease when conventional methods fall short. Moreover, nuclear imaging modalities are able to better characterize lesions using target agents related to specific pathways (e.g. glucose metabolism, cellular proliferation, amino acid transport, lipid metabolism, specific receptor ligands). The clinical presentation of endocrine diseases encompasses a broad spectrum of sign and symptoms. Moreover, endocrine tumors show varying degrees of aggressiveness from well differentiated and indolent to highly aggressive cancers, respectively.

**Rationale:**

With the application of new medicinal radio-compounds and increasingly advanced tomographic imaging technology, the utility of Positron Emission Tomography/Computed Tomography (PET/CT) in the field of endocrine diseases is expanding.

**Aim:**

This review aims to analyze and summarize the primary indications of PET/CT, providing a practical approach for clinicians. A comprehensive literature search on PubMed was conducted to provide an updated overview of the available evidence regarding the use of PET/CT in endocrinology. Within this review, we will discuss the applications of PET/CT, compare different radiopharmaceuticals and highlight the uptake mechanism, excluding neuroendocrine carcinomas from discussion.

**Conclusions:**

PET/CT is a valuable tool in diagnosing and managing endocrine disorders due to its capacity to furnish both functional and anatomical information, facilitate early lesion detection, guide treatment decisions, and monitor treatment response. Its non-invasive nature and precision make it an integral component of modern endocrine healthcare. This review aims to provide physicians with a clear perspective on the role of PET/CT imaging, discussing its emerging opportunities and appropriateness of use in endocrinological diseases.

## Introduction

**Fig. 1:**
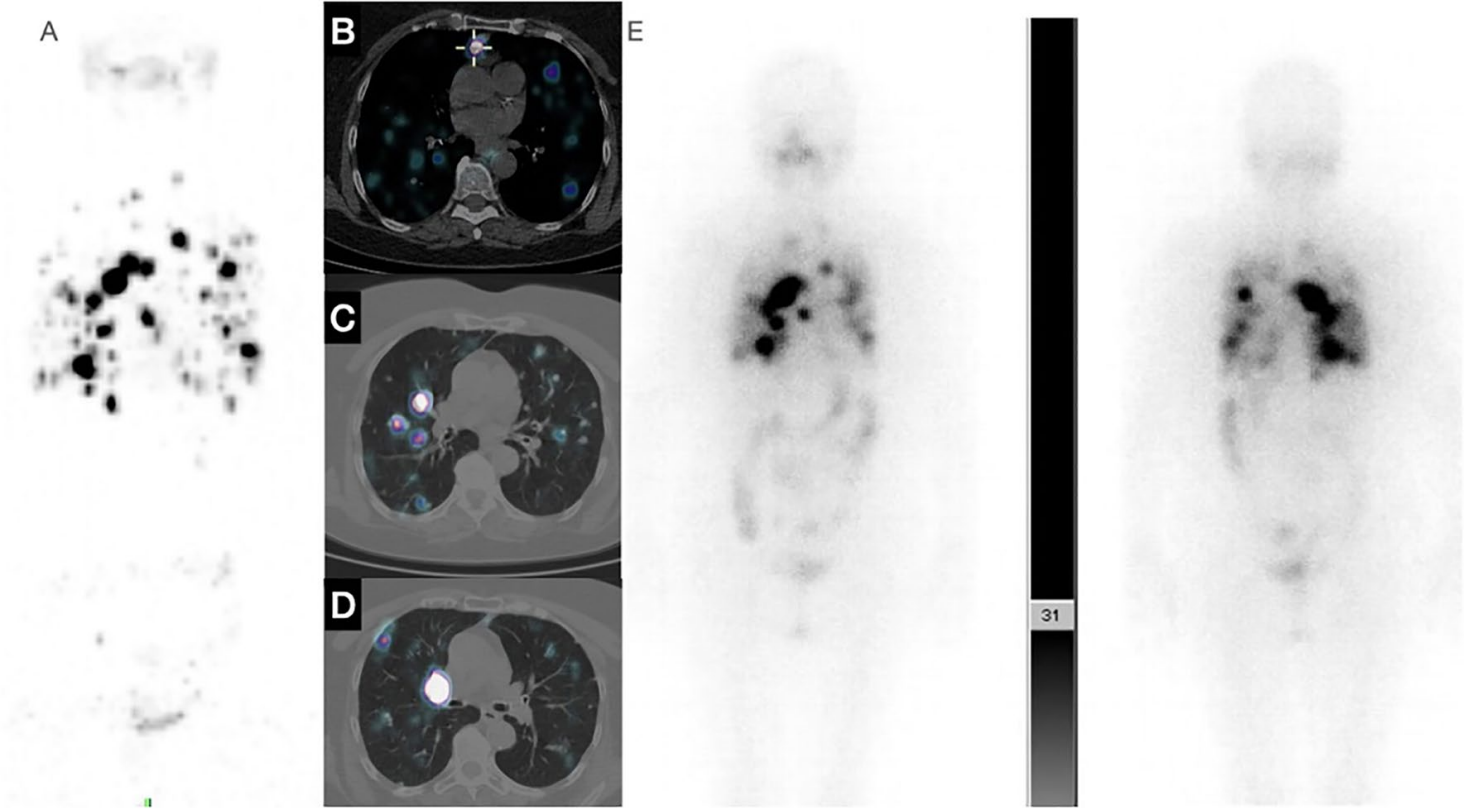
^124^I PET/CT of 63 yo woman with DTC after total thyroidectomy and RAI: (**A**) maximum Intensity Projection (MIP) multiple metastatic findings in the lung and upper mediastinum; axial images show metastatic lymph node in the upper mediastinum (**B**), multiple bilateral lung metastases (**C**–**D**) and a 3 cm solid mass in the right hilum of the lung (**D**); **E** 131-iodine scintigraphy findings after the second radioactive iodine (RAI) treatment: 131-iodine has accumulated in the metastatic tumor in the lungs and in the mediastinal lymph node

Over the last decade, the role of nuclear imaging methods has become increasingly pivotal across a diverse range of clinical conditions, and notably so in the realm of oncology [1]. These imaging techniques have emerged as indispensable tools for precisely assessing the extent of disease, particularly in situations where conventional diagnostic methods may prove inadequate [1]. The realm of endocrine diseases presents a multifaceted clinical landscape, characterized by a wide spectrum of presentations and endocrine malignancies span from indolent to highly aggressive tumors, respectively [2]. This inherent complexity demands innovative and sophisticated approaches to diagnosis and management. The evolving landscape of medical imaging has witnessed remarkable progress, thanks in part to the introduction of novel medicinal radiopharmaceuticals and the continuous refinement of tomographic imaging technology [3]. In this context, Positron Emission Tomography/Computed Tomography (PET/CT) has garnered increasing attention and importance [3]. The integration of cutting-edge radiopharmaceuticals and the advancement of tomographic imaging techniques have substantially expanded the utility of PET/CT within the field of endocrine diseases [4]. The application of PET/CT now extends beyond conventional boundaries, offering healthcare professionals an enhanced ability to delve into the intricate nuances of endocrine disorders. This technology provides a comprehensive and multifaceted perspective on these conditions, enabling clinicians to make more informed decisions regarding diagnosis, treatment planning, response assessment and patient management [1]. As the field of nuclear imaging continues to evolve, PET/CT is poised to play an even more prominent role in addressing the complexities of endocrine diseases, ultimately leading to improved patient outcomes and a deeper understanding of these intricate medical conditions [5]. This article explores the latest advancements and research in the field of PET/CT, highlighting its growing role in endocrine disorders. Additionally, it analyzes the potential of personalized medicine in the field of endocrinology, where PET/CT is poised to make a significant impact (Table 1; Fig. 1).

**Table 1:** PET tracers and clinical application in endocrinology

Radionuclide	Half Life	Production	Traced pathway	Clinical applications	Teragnostic
[18F]F-FDG	109.8 min	Cyclotron	Glucose metabolism (GLUTs)	Oncology: ATC, dedifferentiated DTC, MTC, PC, adrenal malignancies (ACC and metastases)	No
[18F]F-DOPA	109.8 min	Cyclotron	Dopamine synthesis (LAT1)	MTC, pheochromocitomas, paragangliomas, insulinomas	No
[11C]C-methionine	20.04 min	Cyclotron	Amino acid transport and protein metabolism	Oncology: brain tumors (pituitary neoplasms)	No
124I-Na	4.2 days	Nuclear reactor	Iodine metabolism (NIS)	DTC	Yes
[18F]F-TFB	109.8 min	Cyclotron	Iodine metabolism (NIS)	Pre-clinical application DTC	No
[18F]F-FSO3	109.8 min	Cyclotron	Iodine metabolism (NIS)	Pre-clinical application DTC	No
[18F]F-fluorocholine	109.8 min	Cyclotron	Metabolism and turn-over of cell membranes	HPT	No
[11C]C-choline	20.04 min	Cyclotron	Metabolism and turn-over of cell membranes	HPT	No
[68 Ga]Ga-SSRA	67.7 min	Generator	SSRAexpression	NET and pituitary neoplasms	Yes
FAP-targeting tracers	67.7 min	Generator	Activated fibroblast metabolism	Clinical Trials	Yes
[68 Ga]Ga-PSMA	67.7 min	Generator	PSMA-receptor expression	Clinical Trials	Yes
[68 Ga]Ga-PentixaFor	67.7 min	Generator	CXCR4 receptor	PA	Yes
[11C]C-metomidate	20.04 min	Cyclotron	CYP11B enzyme	PA	No

*ATC* anaplastic thyroid carcinoma, *DTC* differentiated thyroid cancer, *MTC* medullary thyroid carcinoma, *PC* parathyroid carcinoma, *ACC* adrenal cortex carcinoma, *HPT* hyperparathyroidism, *NET* neuroendocrine tumors, *PA* primary aldosteronism

### Positron-emitting radiopharmaceuticals and uptake mechanism

**Fig. 2:**
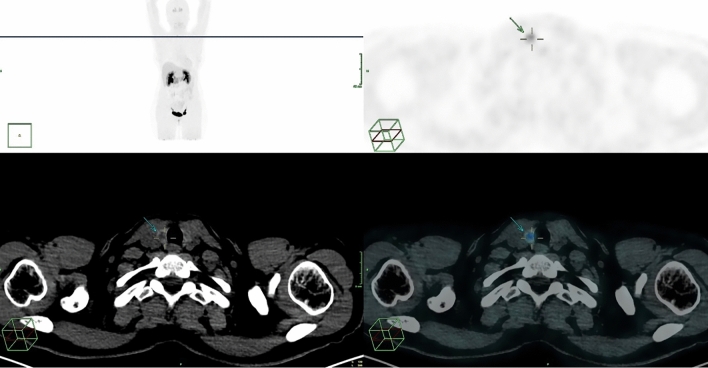
[^18^F]FDOPA PET/CT preoperative staging in a 61 y.o. woman with hypercalcitonemia and mild elevation of serum CEA, showing focal uptake in thyroid nodule in the right lobe; no evidence of pathological lymph nodes. A MTC was diagnosed after total thyroidectomy

Positron-emitting radionuclides find application in diagnostic imaging by emitting two monochromatic photons with an energy of 511 keV following a positron–electron annihilation event. Chemical forms containing positron-emitting radionuclides are generated by cyclotrons [6] (Table 2; Fig. 2).

**Table 2:**
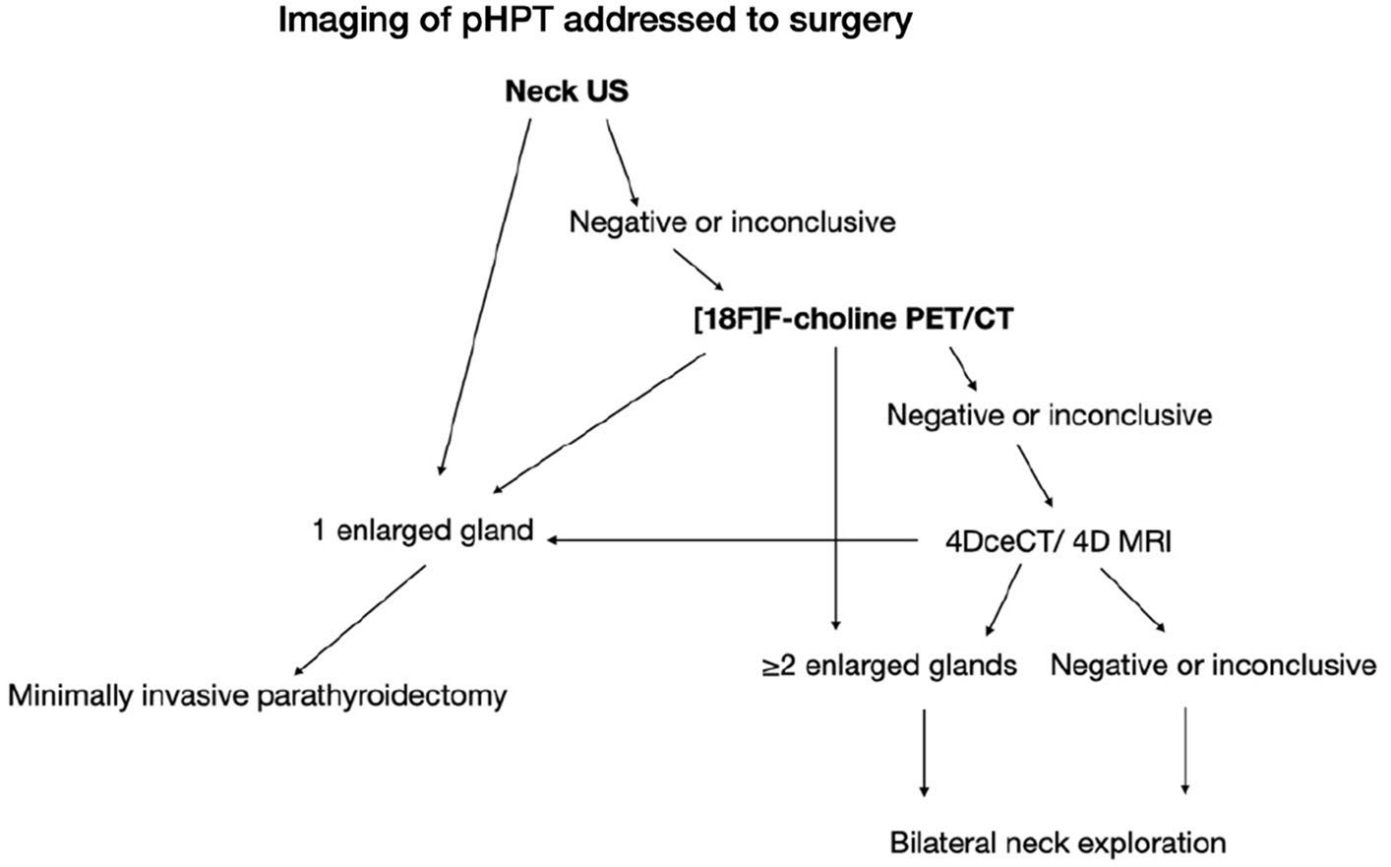
Proposed flowchart algorithm to use PET imaging to guide surgical management

### 2-[^18^F]FDG

**Fig. 3:**
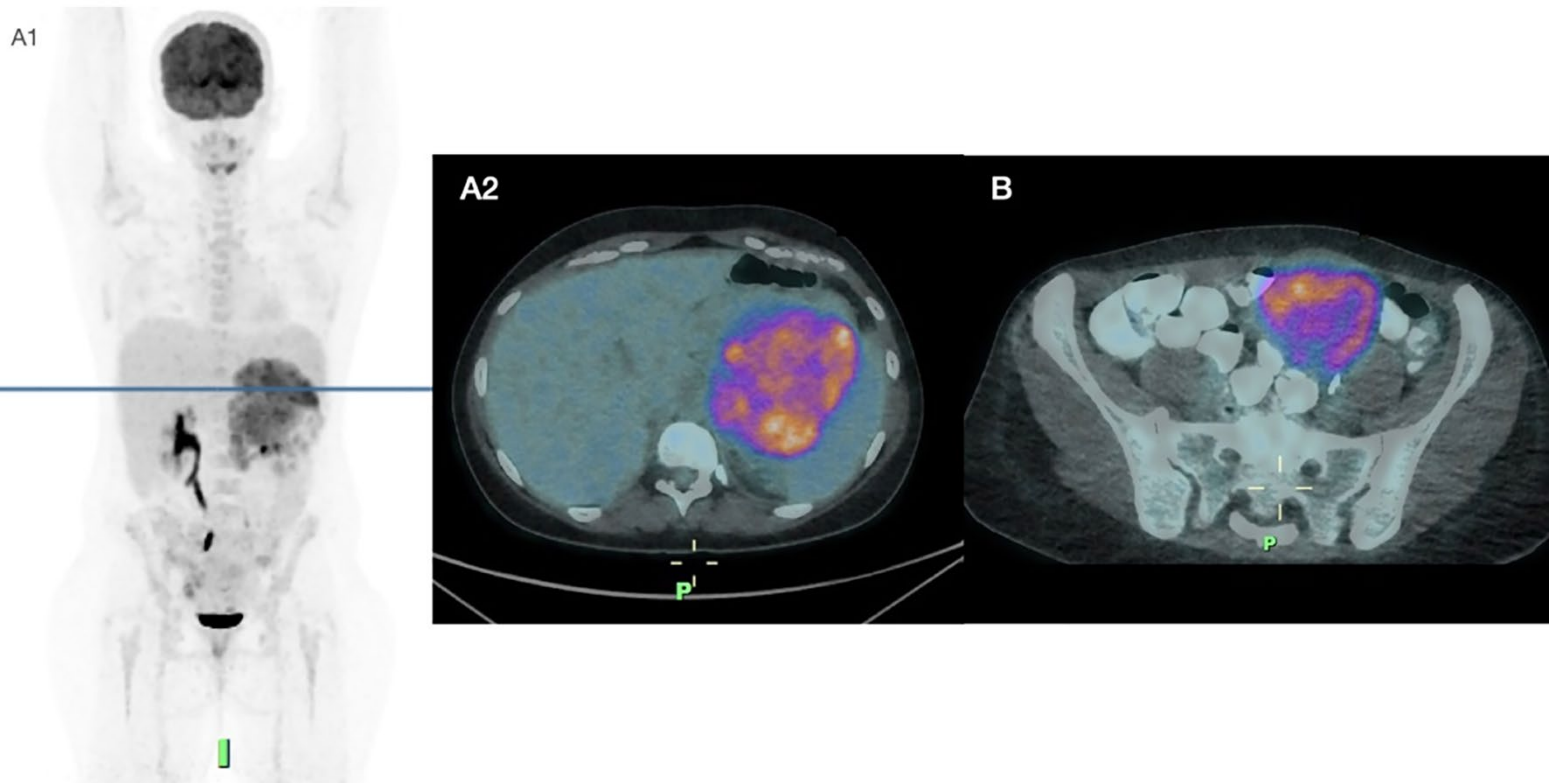
Preoperative staging with [^18^F]F FDG PET/CT(A1) in a 32-year-old patient with a finding of a voluminous left adrenal mass: (A2) axial PET/CT images show an intense and inhomogeneous uptake in the adrenal mass and in some lumbar aortic lymph nodes; after left nephrectosurrenectomy and left lymphadenectomy, a diagnosis of high-grade ACC was made. **B** At follow-up PET scan, during mitotane therapy, was found a peritoneal carcinosis

2-Deoxy-2-[^18^F]fluoro-d-glucose (2-[^18^F]FDG) or [^18^F]FDG is a glucose analogue that enters cells through glucose transporters (GLUTs) and competes with glucose for uptake. When injected intravenously, it quickly spreads through body fluids and is taken up by various tissues through GLUTs, getting trapped inside cells [7]. Its distribution in tumor tissue is proportional to blood flow, and it’s transported into cells via facilitated diffusion by specific glucose transporters, especially GLUT-1. High GLUT-1 levels are associated with increased [^18^F]FDG uptake in human tumors and are also considered a marker of hypoxia, as tumor blood vessels often fail to meet local metabolic needs [7] (Fig. 3).

### [^11^C]choline and [^18^F]F-choline

**Fig. 4:**
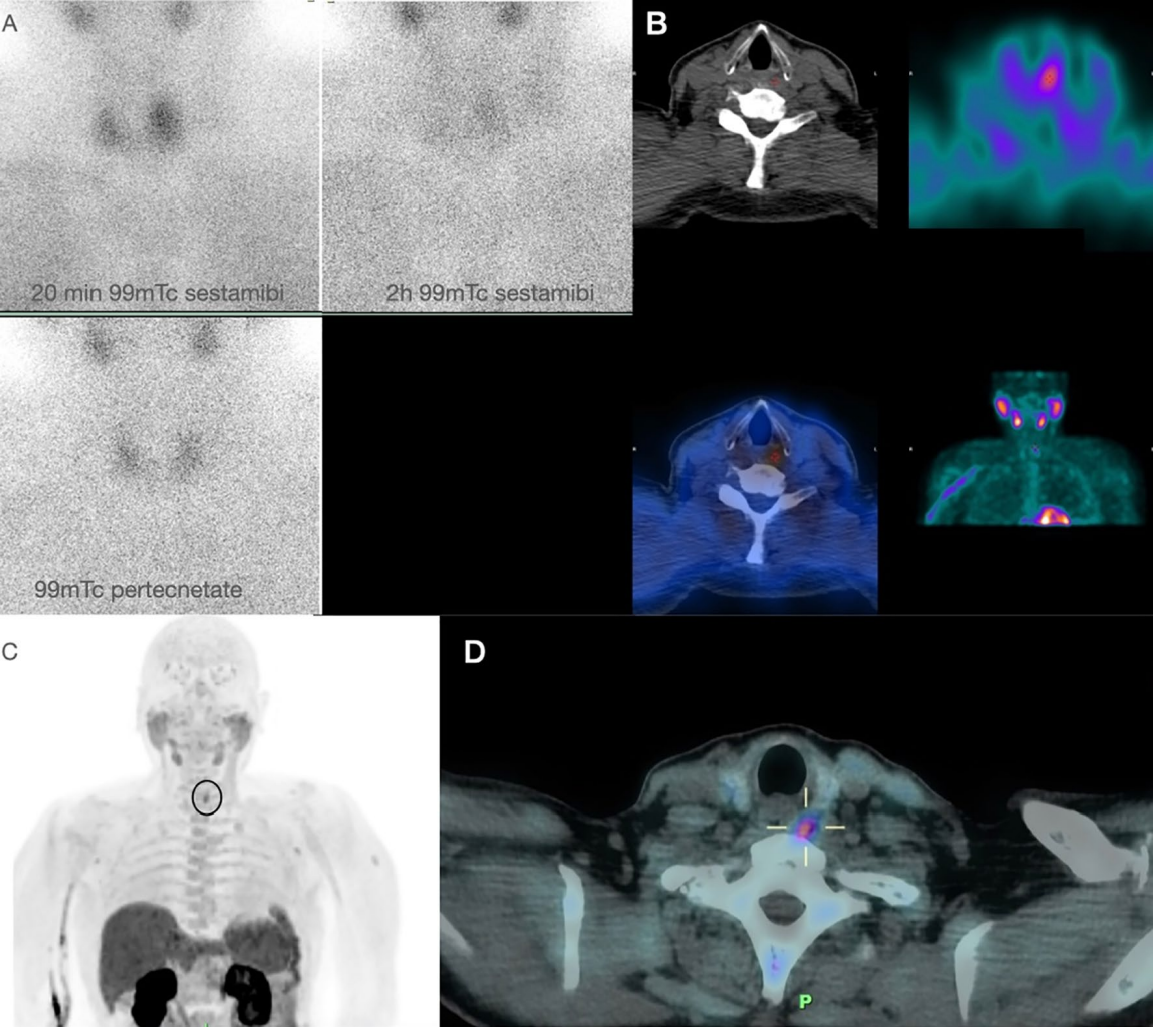
**A** Double tracer ^99m^Tc-sestamibi and ^99m^Tc pertechnetate in 71-year-old patient with primary HPT (PTH 226 pg/ml) and no evidence of enlarged parathyroid on ultrasonography and 4D CT; **B** SPECT/CT shows no findings likely to be an adenomatous parathyroid. **C**–**D** [^18^F]F-choline PET/CT MIP and axial images show the presence of small area of focal hyperfixation at the left upper parathyroid

Choline plays a crucial role in various biological processes, such as the synthesis of phospholipids in cell membranes, methyl metabolism, cholinergic neurotransmission, transmembrane signaling, and lipid-cholesterol transport and metabolism [8]. Choline-based PET tracers are substrates of phospholipid synthesis [4]. Tumor cells have a high demand for choline due to the rapid duplication of cell membranes, and they incorporate choline quickly to support this process [8]. Consequently, levels of choline and phosphorylcholine are elevated in many tumor cells, indicating increased choline uptake and phosphorylation. In slowly proliferating tumors, elevated phospholipid metabolite levels are associated with alterations in choline transport, incorporation, and utilization [9]. [^11^C]choline, biochemically identical to native choline, is commonly used for PET/CT imaging, particularly in patients with prostate cancer and other malignancies that do not exhibit GLUT system overexpression [9]. Physiological uptake of [^11^C]choline is observed in various glands (pituitary, salivary glands, pancreas), as well as in the liver, kidney, bowel, and stomach [8]. However, the short physical half-life of ^11^C (20 min) restricts its use to PET centers with on-site cyclotron facilities [10]. To address this limitation, ^18^F-labeled choline analogs, such as 18F-fluoromethylcholine, have been developed, mirroring the metabolic processing of native choline and serving as commercially available PET imaging agents [10] (Fig. 4).

### ^124^I e other iodine-analogs

Visualization of NIS (sodium-iodide symporter) expression through in vivo molecular imaging has traditionally relied on diagnostic [^123^I or ^131^I] or post-therapeutic whole-body scans using [^131^I] [4]. An alternative long-lived PET radionuclide that is gaining increasing attention for clinical and long-term PET studies is iodine-124 (^124^I), which has biochemical behavior identical to other iodine isotopes, combining tracer specificity with high resolution of PET/CT [5].

The spatial resolution of images using this radionuclide is comparable to that achieved with more conventional PET tracers and its half-life of 4.2 days is suitable for imaging slow physiological processes, aided by the elimination of non-specific radioactivity, therefore appears to be the most promising iodine isotope for individual pretherapeutic dosimetry [5].

Finally, new radiopharmaceuticals are being developed for iodine pathway tracing, including [^18^F]tetrafluoroborate ([^18^F]TFB) and [^18^F]fluorosulfate ([^18^F]FSO3) [13]. From a technical perspective, the development of fluorinated tracers offers advantages such as easier labeling, improved image quality, and high tumor-background contrast in both animal and human studies [13]. Biologically, [^18^F]TFB and [^18^F]FSO3 are analogues of iodine, but they do not undergo the process of organizing iodine in thyroid cells [14]. Furthermore, they offer practical advantages as they use an easily synthesizable radiotracer fluorine-18, which is more accessible and economical than ^124^I [14].

### [^11^C]methionine and [^18^F]FDOPA

In the past, the most widely used amino acid for PET tumor imaging was [^11^C]methionine [4]. Although the precise method of uptake by tumor cells remains somewhat unclear, it likely involves the transport of substrates across the tumor cell membrane [19]. [^11^C]methionine has been used primarily for the study of brain tumors, where the use of [^18^F]FDG can be problematic due to physiologically high uptake in normal gray matter of the brain [4]. The amino acid dihydroxyphenylalanine (DOPA) can be labeled with ^11^C to produce the tracer [^11^C]DOPA or with ^18^F to produce ^18^F-DOPA)[4]. As l-DOPA is the precursor of the neurotransmitters dopamine, norepinephrine and epinephrine, 6-[^18^F]FDOPA ([^18^F]FDOPA) enters the catecholamine metabolic pathway in both the brain and peripheral tissues [4]. In cases of heightened activity of l-DOPA decarboxylase [^18^F]FDOPA PET/CT is a valuable instrument [10]. [^18^F]FDOPA enters cells via amino acid transport systems (LAT1) for large neutral amino acids, which are present in almost all cells [10]. The enzyme aromatic amino acid decarboxylase (AADC) metabolizes 18F-DOPA into 6-18F-fluorodopamine, which in turn can be stored in secretory vesicles by the vesicular monoamine transporter (VMAT), thus effectively becoming trapped within the cell [10]. Clinical applications of PET/CT using [^18^F]FDOPA includes assessing the degeneration of presynaptic dopaminergic neurons, particularly in conditions like Parkinson’s disease [10]. It is also utilized for oncology, in particular imaging brain tumors and visualizing well-differentiated neuroendocrine tumors (NETs) such as medullary thyroid cancer, pheochromocytoma, paraganglioma, and congenital hyperinsulinemic hypoglycemia. Physiologically, [^18^F]FDOPA accumulates in the basal ganglia, liver, pancreas, adrenal glands, gallbladder, biliary tract, kidneys, ureters, and urinary bladder [10].

### [^68^Ga]-DOTA-peptides

The [^68^Ga]-DOTA-peptides ([^68^Ga]Ga-DOTANOC, [^68^Ga]Ga-DOTATOC, [^68^Ga]Ga-DOTATATE) exhibit high expression of somatostatin receptors (SSTR) [11]. Therefore, such PET probes allow the evaluation of the extent of disease and the suitability for peptide receptor radionuclide therapy (PRRT) in patients with differentiated neuroendocrine neoplasms (NETs), as well as the assessment of response, optimization of the treatment sequence, feasibility of PRRT-retreatment, and combination of PRRT with other therapeutic options [11]. In this setting, PET/CT has become an essential tool, since such a heterogeneous group of tumors arising from neuroendocrine cells can arise throughout the body, accurate localization and staging are essential for optimal management [12]. PET/CT has the advantage of detecting even small lesions and assessing their receptor expression in vivo [12].

### PET/CT applications in clinical endocrinology

#### PET/CT in parathyroid disorders

Primary hyperparathyroidism (pHPT) is the third most common endocrine disease, in most cases due to inappropriate secretion of parathyroid hormone by one or more parathyroid adenomas [15]. Minimally invasive parathyroidectomy represents standard of care in pHPT as it can lead to clinical complications (hypercalcemia, kidney stones or osteoporosis) [16], even though asymptomatic at diagnosis in 80% [17]. Precise preoperative identification of parathyroid adenomas is crucial because minimally invasive surgery reduces surgical complications and expedites patients’ recovery therefore preoperative imaging for parathyroid adenomas is of great importance [18]. In secondary hyperparathyroidism (sHPT) surgery is not recommended, while patients with tertiary hyperparathyroidism (tHPT) often undergo total or subtotal parathyroidectomy, therefore the identification of adenomatous glands may be useful to assist surgical planning [19]. Furthermore, persistence or recurrence of HPT after parathyroidectomy occurs in approximately 5–10% of patients [20]. Normal parathyroid glands are rarely detected at cervical ultrasound (US) while enlarged hyperfunctioning ones (i.e. adenoma, hyperplasia) are detectable in a significant proportion of pHPT patients making US the first-line preoperative localization method [21]. However, US sensitivity varies considerably, from 55 to 88%, and depends on factors such as the experience of the examiner, the size of the adenoma, the presence of ectopic lesions, coexisting multinodular goiter, and the presence of enlarged cervical lymph nodes [22]. Second-line nuclear-medicine imaging is challenging because a radiopharmaceutical with specific uptake in hyperfunctioning adenomas is missing, therefore scintigraphy with [^99m^Tc]Tc-sestamibi with Single Photon Emission Tomography/CT (SPECT/CT) is currently in routine use where available, with sensitivity values of 80% according to some authors [23]. In the latest guidelines for parathyroid imaging, the European Association of Nuclear Medicine (EANM) emphasized the prominent role of 99mTc-MIBI as the primary imaging tracer for pHPT, suggesting that the combination of SPECT/CT with cervical US can achieve a remarkable sensitivity of up to 95% to detect functional parathyroid glands [24].

It is important to note that [^99m^Tc]Tc-sestamibi SPECT/CT may show false-positive results in case of benign thyroid nodules (especially oncocytic adenomas) and thyroid malignancies and false-negative results for small, cystic or necrotic parathyroid adenomas [25]. According to literature after negative or inconclusive conventional imaging, several PET radiopharmaceuticals may be useful second line agent in pHPT [25, 26]; [^11^C]methionine and choline-radiolabelled drugs are reliable with good specificity and sensitivity values. According to literature methionine PET/CT showed an overall high sensitivity and positive predictive value (PPV), underscoring its potential for facilitating minimally invasive parathyroidectomy [27]. Furthermore, [^18^F]F-fluorocholine PET/CT may offer an even greater level of diagnostic accuracy, improving the overall detection rate to 97.9% according to some Authors [8, 27, 28].

All in all, [^18^F]F-choline PET/CT is now considered the imaging method of choice in patients with tHPT due to its superior detection capability, also in cases of multiglandular disease and a low rate of localization failure of parathyroid adenoma [8]. Excellent sensitivity values of [^11^C]methionine PET/CT were also reported in the tertiary HPT setting, where multiple lesions were visualized in 57.9% of patients and ectopic lesions in 21.1% of patients [19]. According to Bijnens et al., [^18^F]F-choline PET/CT has proven to be a highly accurate method and obviates the need for extensive exploration in most cases where ultrasound and scintigraphy results do not align or both show negative results [21]. Nonetheless, its application in this context might face limitations because of cost and accessibility factors. These results highlight the promising role of nuclear imaging techniques, particularly [^11^C]choline and [^11^C]methionine PET/CT, in optimizing the precision and efficacy of parathyroid surgery, ultimately benefiting patients through reduced invasiveness and improved treatment outcomes. Further research and clinical evaluation are warranted to fully harness the capabilities of these advanced imaging modalities for enhanced patient care in the field of endocrinology. Table 2 shows a flowchart algorithm to use PET imaging to guide surgical management.

In the context of HPT, a brief mention must be made of brown tumors, abnormal bone repair processes resulting from HPT. The diagnosis of these lytic lesions in nuclear medicine is not uncommon, given their use in the management of both cancer and hyperparathyroidism [29, 30]. According to Jacquet-Francillon et al. [^18^F]F-fluorocholine PET/CT is preferable to [^18^F]FDG PET/CT, [^18^F]F-sodium-fluoride and in bone scintigraphy and it can simulate metastatic disease. The uptake of brown tumor appears reversible after parathyroidectomy, with a variable decrease depending on the type of disease [31].

Moreover, must be taken into account that other radiopharmaceuticals, such as [^18^F]FDG and [^18^F]FDOPA, can lead to false-positive findings in patients with HPT. For example, Terroir et al. report on a 43-year-old patient with MEN 2A suffering from hyperparathyroidism and medullary thyroid carcinoma (MTC). Two focal uptake areas within thyroid with [^18^F]F-DOPA were reported as multifocal MTC and two others in the central compartment of the neck as metastatic lymph nodes of MTC, however, post-operative pathological analysis revealed areas of multifocal intrathyroidal hyperplasia and two parathyroid glands [32]. Therefore, in case of coexistence of multiple endocrine diseases as in MEN 2A, the clinical finding must be carefully analyzed to correctly interpret [^18^F]FDOPA PET/CT.

Causing less than 1% of pHPT, parathyroid carcinoma (PC) is an extremely rare endocrine tumor (0.005% of all tumors) [33]. In 90% of cases, it is functional and secrete extremely high amounts of parathormone (PTH), but the clinical manifestations of PC are polymorphic, making diagnosis complex. The high recurrence rate and distant metastases are challenge with the most common distant metastases located in the lungs and liver. The usefulness of [^18^F]FDG PET/CT in assessing local recurrence and distant metastasis of PC is challenged. In contrast, [^18^F]F-choline PET/CT seems to be able to detect primary parathyroid carcinoma for preoperative localization and allow a one-stop-shop metastasis detection and whole body staging [34].

#### PET/CT in Thyroid Disorders

##### Role of PET/CT in indeterminate thyroid nodules

Thyroid nodules with indeterminate cytology, accounting for 25% of cases, include follicular lesions of undetermined significance or atypia of undetermined significance (Bethesda class III; malignancy risk ranging from 10 to 30%) and follicular neoplasms (Bethesda class IV; malignancy risk ranging from 25 to 40%) [35]. In clinical practice, it is common to repeat fine-needle aspiration biopsy (FNAB) and perform molecular tests in these cases [36]. Nevertheless, several authors are investigating whether certain radiopharmaceuticals, such as [^99m^Tc]Tc-sestamibi and [^18^F]FDG, allow for the evaluation of the biological behavior and aggressiveness of “cold” thyroid nodules. If there is uptake of [^99m^Tc]Tc-sestamibi and/or [^18^F]FDG, the calculated risk of malignancy is approximately 35%. Conversely, in cases of low or absent uptake of [^99m^Tc]Tc-sestamibi and/or [^18^F]FDG, they are considered to have a very low risk of malignancy, demonstrating a high negative predictive value (NPV ~ 84 to 100%) [35–37]. High values of the maximum standardized uptake value (SUV_max_), a semi-quantitative measure reflecting glucose metabolic activity, may be associated with an increased risk of malignancy. However, SUVmax is not a specific marker for malignancies, and therefore, no statistically satisfying specific threshold has been identified to distinguish malignant lesions. In addition, other PET-derived parameters, such as metabolic tumor volume (MTV) and total lesion glycolysis (TLG), have been examined, but the results lack consistent uniformity [35]. Some authors have shown a reduction in unnecessary surgeries thanks to [^18^F]FDG PET/CT [38]. De Koster et al. demonstrated that [^18^F]FDG PET/CT has high sensitivity values and negative predictive value (94.1% and 95.1% respectively) and that it is therefore a reliable tool to exclude malignant tumors and avoid unnecessary diagnostic interventions in thyroid nodules with indeterminate cytology [38].

##### Focal thyroid incidentalomas

Incidentalomas are unexpected lesions discovered incidentally, usually not related to the original clinical indication for [^18^F]FDG PET/CT, and can be either focal or diffuse. In patients without known thyroid pathology, the prevalence of diffuse uptake is 0.1–4.5% and is usually related to benign processes such as chronic thyroiditis, Graves’ disease, while focal thyroid uptake ranges from 0.1 to 4.8% with an average malignancy rate of about 34% [39]. However, almost two thirds of focal [^18^F]FDG uptakes within the thyroid gland are related to benign diseases [39]. While focal thyroid uptake on [^18^F]FDG PET/CT should always be considered in the context of the possibility of an aggressive tumor, the majority of such instances are not linked to malignant conditions. Many studies evaluated the usefulness of different parameters to differentiate malignant from benign thyroid lesions, with controversial results [39]. Higher SUVmax are more likely to be malignant but to date no statistically significant data are available and a safe threshold has not been identified. For instance, Hurthle and follicular adenomas have higher SUV_max_ compared with other benign conditions [39]. Guidelines from various thyroid associations recommend ultrasound-guided fine-needle cytology (FNAC) for patients presenting with a FDG focal increase uptake, if ever performed or clinically appropriate [40]. Although these guidelines are clear and straightforward, they can sometimes potentially lead to overdiagnosis and overtreatment, impacting the quality of life of these patients [40]. These findings highlight the importance of conducting incidentaloma evaluations within the context of the patient’s medical history.

##### Differentiated thyroid cancer

Molecular imaging plays an important role in the analysis and treatment of various types of thyroid cancer [41]. Differentiated thyroid carcinoma (DTC) is the most prevalent endocrine tumor and typically has a favorable prognosis, except when it becomes refractory to radioiodine therapy (RIT). Identifying cases with a poor prognosis usually occurs after multiple RITs, emphasizing the importance of early and dependable predictive tools. Matsuo et al. investigated whether [^18^F]FDG PET/CT, when combined with initial RIT, could identify early-stage patients with a poor prognosis among those with high-risk DTC [41]. Patients who tested positive for [^18^F]FDG PET/CT had a significantly worse prognosis than those who were negative for initial RIT. This demonstrates that [^18^F]FDG PET/CT plays an important role in both the diagnosis and prognostic prediction of RIT-refractory disease in DTC patients [41].

After surgery and radioiodine (RAI) therapy, monitoring of most patients with DTC is based on serum tumor markers (thyroglobulin and thyroglobulin antibodies) and neck ultrasound, with additional imaging required when a disease spread is suspected [42]. [^18^F]FDG PET/CT proves valuable when there is a suspicion of non-iodine-avid disease, especially in scenarios involving a negative radioactive iodine whole-body scan (WBS) alongside elevated thyroglobulin (Tg) levels or Tg levels that do not align with the WBS findings [38]. Current guidelines suggest considering [^18^F]FDG PET/CT when stimulated thyroglobulin > 10 ng/mL, although even with thyroglobulin levels < 10 ng/mL [^18^F]FDG PET/CT is positive in 10–20% of cases [43]. Moreover, a rapid thyroglobulin doubling time (i.e., less than 1 year) has been independently linked to positive [^18^F]FDG PET/CT results in cases of biochemical relapse [44]. The integration of radioiodine imaging with [^18^F]FDG PET/CT can enhance the effectiveness of subsequent radioiodine treatments and provide insights into alternative strategies, including surgical interventions, external beam radiotherapy or systemic therapies [44]. Specifically, [^18^F]FDG PET/CT is useful to identify the aggressiveness of the disease, conduct a complete distant staging and plays a crucial role in cases of DTC refractory to radioiodine therapy, poorly differentiated tumors and anaplastic carcinomas [43].

[^124^I] PET/CT is a promising method in the management of thyroid cancer patients, by the capability in developing tailored treatment plans, calibrating the therapeutic dose and avoiding ineffective therapies. [^124^I] PET/CT has a higher sensitivity than [^131^I] WBS in detecting secondary DTC lesions, representing a possible tool for personalized dosimetry [45]. The [^124^I] PET/CT allows dosimetry, meaning it identifies the absorbable dose by each lesion and can predict the response to therapy by identifying lesions that may not reach the therapeutic target [45]. Currently, there is a lack of substantial data demonstrating that ^131^I therapy guided by dosimetry yields superior outcomes, including improved disease-free survival and reduced adverse events, compared to empirical therapy [43]. This is especially crucial in pediatric cases. Dosimetry has limitations, so currently, according to the literature, the only significant prognostic factor for survival and predictive factor for treatment response is [^18^F]FDG PET/CT [43].

Recent studies comparing [^18^F]TFB and [^124^I]NaI PET/CT in patients with DTC have shown that the distribution of tracers and the detection rate are similar [13]. ^124^I is retained more in residual thyroid tissue than [^18^F]TFB, probably due to the organization of iodide in thyroid cells, conversely [^18^F]TFB PET was superior in detecting metastases [13].

Fibroblast activating protein (FAP) is overexpressed by cancer-associated fibroblasts of several tumors. Some authors have shown that PET/CT, with a relatively recent tracer that detects the expression of FAP inhibitor (FAPI), in DTC patients with increased Tg or anti-Tg antibodies has diagnostic performance comparable to that of [^18^F]FDG PET/CT, in fact, can be used in patients with inconclusive results [^18^F]FDG PET/CT [46, 47]. In some advanced DTC RAI-R cases who have had disease progression despite undergoing standard treatment regimens, radiopharmaceuticals that enable radioreceptor therapy, such as SSA-tracers and newer radiopharmaceuticals, prostate-specific membrane antigen (PSMA), and FAP-targeting tracers, could be considered [48]. However, their precise role remains uncertain and it is imperative to conduct well-designed studies to evaluate their potential benefits in the most challenging cases [48].

Anaplastic thyroid carcinoma (ATC) is an undifferentiated form of thyroid cancer, is not responsive to RAI and has a poor prognosis, with a median overall survival of 5–6 months and a survival rate of 20% [49]. The use of [^18^F]FDG PET/CT is indicated in the staging phase to accurately evaluate possible surgery and the extent of the disease, which could help guide therapeutic decisions. American Thyroid Association guidelines recommend the use of tyrosine kinase inhibitors, but currently, imaging response predictors are lacking [49].

##### Medullary thyroid cancer

Medullary thyroid carcinoma (MTC) originates from the parafollicular C cells of the thyroid gland and correct staging and risk assessment are crucial to choose the most appropriate therapeutic approach [50]. With regard to MTC, the most appropriate use of [^18^F]FDOPA and [^18^F]FDG PET/CT depends on serum calcitonin and carcinoembryonic antigen (CEA) levels [51]. In patients with suspicious MTC recurrence with serum calcitonin > 150 pg/mL or calcitonin doubling time < 1 year the first line use of [^18^F]FDOPA allows a detection rate of 66% per patient and 71% per lesion) [52].

There is currently insufficient evidence to recommend PET/CT with other radiopharmaceuticals than [^18^F]FDOPA for staging MTC [51]. However [^18^F]FDG may characterize less differentiated lesions (as observed in patients with predominant increase in serum CEA compared to CT) informing changes in therapy lines. As the very least, somatostatin analogs (SSA) are also proposed for MTC restaging but their sensitivity and accuracy are significantly lower to both [^18^F]FDOPA and [^18^F]FDG, respectively [50]. Several experimental nuclear medicine therapeutic options are currently being evaluated in metastatic MTC [52]. Further data are needed to evaluate the efficacy, toxicity and role of these therapeutic options in the management of patients with MTC [53].

Furthermore, it is essential to highlight that there are promising theranostic radiopharmaceuticals for the management of metastatic MTC, in fact emerging therapies using beta- or alpha-emitting radioligands may provide benefits for carefully selected cases with significant expression of specific molecular targets, such as FAPI PET/CT [47]. In a very recent study, [^68^Ga]Ga-DOTA.SA.FAPi was significantly more sensitive than [^68^Ga]Ga-DOTANOC PET/CT in the detection of lung nodules, liver metastases, bone metastases and pleural metastases, uptake values and tumor-to-background ratio were higher with [^68^Ga]Ga-DOTA.SA.FAPi [54].

#### PET/CT in Adrenal Disorders

Adrenal incidentaloma is a term used to describe focal adrenal lesions discovered unexpectedly during abdominal imaging conducted for reasons unrelated to adrenal evaluation (prevalence of about 4–10%) [55]. In patients without a known malignancy, the likelihood of these incidentalomas being cancerous is generally low and are often asymptomatic [55].

There are differing viewpoints in the medical literature regarding the follow-up imaging of adrenal incidentalomas, though current guidelines from the Association of Clinical Endocrinologist and American Association of Endocrine Surgeons recommend evaluation of all adrenal incidentalomas to exclude presence of hyperfunctioning lesion [56]. This evaluation involves a combination of imaging, biochemical assessments, and clinical evaluation by an endocrinologist [56].

[^18^F]FDG PET/CT is effective in identifying hypermetabolic primary cancers (i.e. adrenal carcinoma), metastatic lesions and hyperfunctioning tumors such as pheochromocytoma [57]. Therefore, it is a valuable imaging method that can provide additional information about the nature of a mass, allowing differentiation between benign and malignant lesions. Malignant lesions typically present with a higher SUVmax than benign lesions, with sensitivity ranging from 93 to 100% [57].

A recent meta-analysis aimed to evaluate the diagnostic value of [^18^F]FDG PET/CT in distinguishing between benign and malignant adrenal tumors, particularly in cases of adrenal incidentalomas or those discovered during staging or follow-up of cancer patients [59]. The pooled sensitivity was 87.3% and the pooled specificity was 84.7%, hence [^18^F]FDG PET/CT has good diagnostic accuracy in the characterization of adrenal tumors, although the existing literature has limitations, particularly in the case of adrenal incidentalomas [59]. Some authors have reported that adrenal incidentalomas are relatively common, occurring in approximately 12% of patients undergoing imaging for esophageal cancer [58]. This finding is significant because it can change the therapeutic approach from curative to palliative due to the impact on the oncological prognosis [58]. Interestingly, the majority of these patients had adenocarcinoma (15.9%) and the identification of adrenal incidentalomas led to stage migration in 68.2% of cases [58].

Furthermore, new PET radiotracers have emerged that target specific components of the adrenal cortex, such as CYP11B enzymes (e.g., Iodometomidate or IMAZA) or CXCR4 receptors (e.g., PentixaFor) that are particularly valuable for the diagnosis of tumors originating from the adrenal cortex [60].

According to some authors, [^68^Ga]Ga-PentixaFor PET/CT has good diagnostic accuracy in patients with primary aldosteronism. This noninvasive imaging technique may be considered an alternative to invasive adrenal vein sampling for some patients with primary aldosteronism [61].

In another recent prospective study, [^11^C]metomidate (MTO) PET/CT was found to enable the non-invasive diagnosis of unilateral primary aldosteronism and was comparable in accuracy to adrenal vein sampling in predicting the success of adrenalectomy[62].

A recent study, involving a 62-year-old patient with adrenocortical carcinoma (ACC), demonstrated that [^18^F]FDG PET/CT was more effective than [^68^Ga]Ga-FAPI-04 PET/CT [63]. The advantage of FAPI and SSA tracers is to act as anticancer therapies when used in therapeutic equivalents [63].

Adrenal metastases are relatively common and often occur as secondary tumors originating from primary cancers in organs such as the lung, breast, kidney, and melanoma [63]. They are considered the second most common adrenal lesion following adenomas. Adrenal metastatic lesions can be bilateral in nearly half of the cases. Individuals with known malignancies are more likely to have adrenal metastases[63]. [^18^F]FDG PET/CT are effective tools for distinguishing between benign and malignant adrenal lesions, primarily because metastatic lesions typically exhibit higher [^18^F]FDG uptake when compared to the liver. Nonetheless, in certain cases, benign lesions may display similar features, potentially leading to false-positive results [63].

Adrenal metastases typically present as irregular, round lesions with a varied internal texture [64]. Alternatively, they can manifest as an enlarged gland with indistinct borders. On unenhanced CT scans, these lesions are often depicted with an attenuation value exceeding 10 Hounsfield Units (HU) [64]. In patients with established malignancies, adrenal lesions larger than 3 cm in size, or masses lacking hemorrhage or calcification with an attenuation of approximately 43 HU, strongly suggest metastatic involvement. Some highly vascular metastatic lesions may resemble pheochromocytomas in CT images [64].

Pheochromocytomas are rare tumors originating from neural crest cells, with reported incidence rates ranging from 0.04 to 0.21 per 100,000 person-years [65]. They can be bilateral, occur in children, and occasionally exhibit malignancy. Paragangliomas, closely related tumors, are distinguished by their extra-adrenal location and can be associated with familial syndromes [65]. Pheochromocytomas typically present with symptoms related to the excessive secretion of catecholamines. Diagnosis can be confirmed through serum or urine metanephrines measurement [55]. Imaging plays a crucial role in identifying and assessing these tumors. In patients with typical clinical symptoms and elevated catecholamine levels, contrast-enhanced CT or MRI is used to evaluate the adrenal glands and the entire retroperitoneum. As occasional finding CT without contrast or MRI can help distinguish pheochromocytomas from lipid-rich masses (with CT attenuation < 10 HU)[66].

Pheochromocytomas and paragangliomas have varying imaging characteristics and may not always exhibit typical features [67]. They are generally larger than adenomas but smaller than adrenocortical carcinomas, appearing as ovoid-shaped masses of 1–10 cm with heterogeneous early enhancement. CT attenuation is typically around 35.9 HU, with possible necrosis, fibrosis, and calcifications. The presence of intracellular fat rules out pheochromocytoma. Contrast washout is slower compared to adenomas but may overlap, making interpretation challenging [67].

While conventional imaging methods have their merits, advanced PET tracers have emerged as promising tools in the detection and localization of pheochromocytomas and paragangliomas [68]. The diagnostic sensitivity of [^18^F]FDG PET/CT is variable, being higher in malignant pheochromocytomas compared to benign ones (82% versus 58%) [60]. This variation is expected due to the increased glucose metabolic activity observed in more aggressively growing tumors. However, it’s important to note that, as of now, [^18^F]FDG PET/CT is not a standard component of the typical diagnostic protocol for pheochromocytoma [60].

Superior diagnostic performance has been reported for PET/CT using [^18^F]FDOPA compared to [^18^F]FDG [66]. [^18^F]FDOPA shares the same specificity as Metaiodobenzylguanidine (MIBG) for chromaffin tissues, while also benefiting from the superior spatial resolution and improved signal-to-noise ratios offered by PET/CT imaging compared to single-photon imaging [66]. Moreover, the inherent hybrid nature of PET/CT imaging enables precise anatomical localization of the lesions detected using the PET tracer [67]. Existing literature data suggest that [^18^F]FDOPA PET/CT outperforms MIBG scintigraphy in terms of both sensitivity and specificity. This advantage is particularly pronounced in the case of extra-adrenal pheochromocytomas located in the head and neck region [68].

In addition, other PET tracers like [^18^F]F-Fluorodopamine, [^11^C]hydroxy-ephedrine and [^11^C]epinephrine are currently undergoing clinical validation[69]. SSA PET tracers and other nuclear medicine techniques are used in certain cases, particularly when there are specific genetic mutations or tumor recurrence [70, 71].

#### PET/CT in pituitary disorders

Pituitary disorders cover a wide range of conditions affecting the pituitary gland, with benign adenomas being the most common [72]. These adenomas can lead to significant health problems, including vision loss and hormone overproduction, necessitating a combination of surgery, radiotherapy, and medical treatment [72].

Traditional imaging methods like magnetic resonance imaging (MRI) have been the standard for visualizing pituitary adenomas. However, emerging evidence suggests a potential role for PET/CT in specific cases, particularly in identifying germ cell tumors and differentiating recurrent pituitary tumors from scar tissue [53].

For non-functioning pituitary adenomas (NFPA), PET/CT with SSA outperforms [^18^F]FDG PET/CT, especially in cases of recurrent or residual disease [72]. [^11^C]methionine PET/CT also shows promise in distinguishing between residual/recurrent adenomas and normal pituitary remnants [72]. Occasionally, [^18^F]FDG PET/CT may incidentally detect NFPA, even though it isn’t the primary tool for evaluating primary pituitary pathology [72].

At present, the diagnosis of Cushing’s disease (CD) remains a challenge, since microadenomas are difficult to visualize [73]. Different radiopharmaceuticals have been explored for localizing corticotroph adenomas, with varying results [73]. A recent meta-analysis illustrated that MRI in 25% of cases is negative or inconclusive; [^11^C]methionine showed higher pituitary adenoma detection than [^18^F]FDG PET-CT (87% vs. 49%) [74]. In addition, there are some works that demonstrated detected 100% detection rates for [^18^F]Fluoroethyl-L-tyrosine (FET), [^68^Ga]Ga-DOTA-TATE, and [^68^Ga]Ga-Corticotrophin Releasing Hormone (CRH). [^68^Ga]Ga-DOTA-CRH, but based on single studies [74]. Importantly, corticotrophic microadenomas are among the most difficult subtypes of pituitary tumors to visualize [75]. Another new radiopharmaceutical, [^68^Ga]Ga-PentixaFor (for imaging the CXCR4 chemokine receptor), was able to distinguish between ACTH-independent and ACTH-dependent Cushing’s disease [76]. In Langerhans cell histiocytosis (LCH), [^18^F]FDG PET/CT has high sensitivity for assessing pituitary involvement, while [^68^Ga]Ga-SSA PET exhibits intense physiological pituitary uptake, limiting its accuracy in evaluating localized pituitary pathology [76]. There is a growing preference for somatostatin or dopamine receptor ligands and amino acid uptake ligands. These findings highlight the potential of molecular imaging to guide treatment decisions for residual pituitary tumors, especially in cases with difficult MRI interpretations or recurrent disease [72]. Moreover, innovative approaches to distinguish between adenomatous tissue and the normal gland, like comparing images obtained with different radiotracers, and increasing confidence in identifying pathological foci, such as through subtraction imaging, have been proposed. Molecular imaging is likely to continue gaining importance in the management of pituitary tumors, similar to its role in other endocrine disorders.

PET/CT has revolutionized the field of medical imaging, allowing for the simultaneous visualization of anatomical and functional information. In recent years, advancements in PET/CT technology, the development of new radiopharmaceuticals, and innovative hybrid imaging techniques have expanded its applications in endocrinology. The evolution of PET/CT devices has led to improved image resolution, reduced scan times, and enhanced patient comfort. Notably, the development of time-of-flight (TOF) PET/CT provides more precise localization of radioactive emissions, resulting in improved image quality. Additionally, digital PET detectors offer enhanced sensitivity, enabling lower radiation doses and shorter scan times while maintaining diagnostic accuracy.

The discovery and development of new radiopharmaceuticals have broadened the scope of PET/CT applications in endocrinology, opening up new theranostic possibilities, such as radiotherapeutics targeting FAPI or PSMA. The integration of PET/CT into the realm of personalized medicine holds particular promise in endocrinology. By combining functional information from PET/CT with genomic data and clinical parameters, physicians can tailor treatment strategies for individual patients. This approach is especially relevant in thyroid cancer, where risk stratification based on [^18^F]FDG uptake and genetic markers can guide the selection of appropriate therapies, including radioactive iodine treatment.

In conclusion, while PET/CT offers significant advantages in diagnosing and managing endocrine disorders, it also raises complex ethical considerations related to resource allocation, accessibility, radiation exposure, informed consent, overdiagnosis, privacy, clinical utility, research ethics, and healthcare costs. Healthcare professionals, policymakers, and ethicists must collaborate to address these ethical challenges, ensuring that the use of PET/CT in endocrinology is guided by principles that prioritize patient well-being, equity, and ethical healthcare delivery.
